# Characterization of the intestinal microbiota in MSM with HIV infection

**DOI:** 10.1186/s12866-024-03351-z

**Published:** 2024-06-03

**Authors:** Yuansheng Fu, Susu Ke, Gan Tang, Qisheng Guo, Qian Guo, Ziwei Wang, Ruixue Leng, Yinguang Fan

**Affiliations:** 1 Anhui Provincial Center for Disease Control and Prevention, Hefei, Anhui 230601 China; 2 Department of Epidemiology and Biostatistics, School of Public Health, Anhui Medical University, Hefei, China; 3 Inflammation and Immune Mediated Diseases Laboratory of Anhui Province, Hefei, Anhui China

**Keywords:** HIV, Intestinal microbiota, ART, MSM

## Abstract

**Background:**

HIV-infected persons demonstrate notable disturbances in their intestinal microbiota; however, the impact of intestinal microbiota on HIV susceptibility in men who have sex with men (MSM), as well as the effects of HIV and antiretroviral therapy (ART) on their gut microbiota, remains under active study. Thus, our research focuses on clarifying the distinctions in intestinal microbiota composition among uninfected MSM and non-MSM healthy controls, investigating the alterations in early-stage intestinal microbial communities following HIV infection, and assessing how ART affects the intestinal microbiota.

**Methods:**

This study enrolled four participant groups: uninfected MSM, Recent HIV-1 infection (RHI) MSM, MSM on ART, and non-MSM healthy controls, with 30 individuals in each group. We utilized 16S ribosomal DNA (16S rDNA) amplicon sequencing to analyze fecal microbiota and employed Luminex multiplex assays to measure plasma markers for microbial translocation (LBP, sCD14) and the inflammatory marker CRP.

**Findings:**

Comparing uninfected MSM to non-MSM healthy controls, no substantial variances were observed in α and β diversity. Uninfected MSM had higher average relative abundances of Bacteroidetes, *Prevotella*, and *Alloprevotella*, while *Bacteroides*, Firmicutes, and *Faecalibacterium* had lower average relative abundances. MSM on ART had lower intestinal microbiota diversity than RHI MSM and uninfected MSM. In MSM on ART, *Megasphaera* and *Fusobacterium* increased, while *Faecalibacterium* and *Roseburia* decreased at genus level. Additionally, treatment with a non-nucleoside reverse transcriptase inhibitor (NNRTI) led to significant alterations in intestinal microbiota diversity and composition compared to RHI MSM. The random forest model showed that HIV infection biomarkers effectively distinguished between newly diagnosed HIV-infected MSM and HIV-negative MSM, with an ROC AUC of 76.24% (*95% CI*: 61.17-91.31%).

**Conclusions:**

MSM showed early intestinal microbiota imbalances after new HIV infection. MSM on ART experienced worsened dysbiosis, indicating a combined effect of HIV and ART. NNRTI-based treatment notably changed intestinal microbiota, suggesting a potential direct impact of NNRTI drugs on intestinal microbiota.

**Supplementary Information:**

The online version contains supplementary material available at 10.1186/s12866-024-03351-z.

## Introduction

Globally, Human Immunodeficiency Virus (HIV) infection is still a serious public health risk, causing Acquired Immunodeficiency Syndrome (AIDS) in those affected, which was first identified in the early 1980s [1, 2]. By the close of 2021, 38 million had HIV globally, with 1.5 million new infections were reported within that year [3]. As of 2020, China had reported approximately 1.053 million cases of HIV/AIDS, with a cumulative death toll of 351,000. While the epidemic demonstrated an overall low prevalence pattern, it continued to manifest a high prevalence rate within specific population segments [4].

In recent years, research focusing on men who have sex with men (MSM) has garnered increasing attention due to its significance of sexual health, and social dynamics. MSM are acknowledged as a population at high risk for HIV transmission. The United Nations and Malaysia’s Health Ministry: MSM face 27 times higher HIV risk than general population [5, 6]. In recent years, in China, the reported cases of HIV/AIDS have shown a significant increase. It rose from 0.2% in 2000 to 25.5% in 2017 [7]. A Chinese meta-analysis founded: the HIV infection rate was 5.7% (95% CI: 5.4–6.1%) among MSM [8]. Between 2005 and 2015, data from Chinese sentinel surveillance shows a notable increase in HIV prevalence among MSM, rising from 1.4% to 8.0% [9].

The intestinal microbiota is vital for both intestinal balance and the overall health of the host [10]. This community, which includes bacteria, fungi, viruses, and other microorganisms, is now recognized to exceed a staggering count of over 4 × 10^13^ microbial cells [11]. Intestinal microbiota is influenced by a complex interplay involving host-related factors, environmental exposures, and lifestyle choices, contributing to a dynamic ecosystem within the gut. These influences encompass various determinants, including dietary patterns, age-related variations, environmental exposures, and other key elements. As a result, significant variations in the intestinal microbiota composition among different population groups [12, 13]. Maintaining intestinal balance, supporting immune function, and regulating energy metabolism of these are vital functions attributed to the intestinal microbiota, as evidenced by numerous studies. Imbalances in the intestinal microbiota can cause changes in intestinal barrier function, signaling pathways, and host metabolism. These changes are connected to diseases like obesity, diabetes, cardiovascular issues, and autoimmune disorders [14, 15]. In recent research, it has been demonstrated that the intestinal microbiota is not only crucial in the immunopathogenesis of HIV but also in the development of chronic comorbidities linked to the virus [16, 17]. Surveys confirm differences in intestinal microbiota between men who have sex with women (MSW) and MSM, irrespective of HIV status [18, 19]. HIV infection is linked to dysbiosis, decreased α diversity and increased β diversity in the intestinal microflora [10, 20, 21]. In a study [22], elevated CCR5 levels, an HIV co-receptor, were observed on intestinal CD4^+^ T cells. This suggests that MSM-related intestinal microbiota might pose a risk for HIV transmission. It’s still uncertain if the intestinal microbiota linked to MSM heightens susceptibility to HIV.

This study is based on the sequencing of 16S ribosomal DNA (16S rDNA) amplicons, aiming to compare the intestinal microbial composition and diversity among different groups, including uninfected MSM, recent HIV-1 infection (RHI) MSM, MSM on antiretroviral therapy (ART), and non-MSM healthy controls.

Additionally, we aim to investigate the associations between plasma microbial translocation markers, inflammatory factors, and the intestinal microbiota, exploring potential connections. We analyze the gut microbiota of MSM, identifying unique microbial communities specific to this group. Additionally, we investigate the impact of HIV and ART on their gut microbiota. This research provides a scientific foundation for implementing interventions to modulate the intestinal microbiota, ultimately enhancing immune reconstitution and mitigating disease progression among HIV-infected individuals.

## Methods

### Study design and subjects

The study, a cross-sectional analysis, involved MSM and the general population who lived in Hefei, China, between January and June 2021. Three MSM cohorts were prospectively recruited from the Hefei Qingwei Health Service Center (a community-based organization): (1) HIV-uninfected MSM; (2) RHI MSM: persons potentially infected in the past three months and ART drug-naïve; (3) MSM living with HIV on ART/ART-treated HIV-infected MSM: treatment with at least three ART regimens for ≥ 5 months prior to study entry. The criteria for diagnosing HIV infection are available in the provided literature [23]. After matching the three MSM cohorts for sex, age, and geographic location, the HIV-negative non-MSM population was chosen as healthy controls.

Inclusion criteria: (1) no antibiotics or probiotics in 1 month; (2) 18–60 years old; (3) no gastrointestinal disorders such as peptic ulcer, Crohn’s disease, gastrointestinal surgery, etc.; (4) no history of serious cardiac, hepatic, renal, or psychiatric disease; and (5) no autoimmune diseases.

### Ethics statement

The research protocol, endorsed by Anhui Medical University, was executed in compliance with the Helsinki Declaration guidelines. All subjects gave informed consent for sample collection and analysis.

### Date collection

General demographic information was collected through a questionnaire. Clinical data on MSM on ART and RHI MSM were gathered from the Hefei Center for Disease Control and Prevention database. Literature based on foods that may affect the microbiota [24], a short food frequency questionnaire was developed to assess all participants’ dietary profiles. No significant differences in the consumption patterns of grains, vegetables, fruits, or meat were observed among the four groups (data not shown).

### Assessment of microbial translocation markers and inflammatory cytokines

For all participants, blood samples were taken at recruitment, and plasma was stored at -80 °C after separation. Plasma levels of markers of microbial translocation including lipopolysaccharide-binding protein (LBP) and soluble CD14 (sCD14) and inflammation factor (C-reactive protein (CRP)) were measured using a Luminex Human Discovery Assay (R&D Systems).

### Extraction and sequencing

DNA extraction and 16S rDNA sequencing followed cited protocols [25]. Based on the V3-V4 hypervariable region of 16S rRNA, the extracted genomic DNA was amplified by polymerase chain reaction (PCR) using primer pairs 515 F (GTGCCAGCMGCCGCGGTAA)and 806R (GGACTACHVGGGTWTCTAAT). Subsequently, the obtained 16S rRNA gene amplicons were extracted, purified, and quantified. The spliced Tags were clustered into operational taxonomy units (OTUs) at 97% sequence similarity using USEARCH software (v7.0.1090). The RDP classifier Bayesian algorithm was used to taxonomically annotate representative sequences of OTUs with 97% similarity (compare to the RDP database (http://rdp.cme.msu.edu/)) and to count the community composition of each sample at each taxonomic level of the kingdom phylum family genus species.

### Bioinformatics analysis

FLASH(v1.2.11) [26] software for sequence splicing. Clustering was performed using UPARSE (v7.0.1090) [27] and Gold Database (v20110519), chimeras removed with UCHIME (v4.2.40) [28] and Gold Database (v20110519).

This study utilized MOTHUR (v1.31.2) software to perform alpha diversity calculations including Shannon index, Chao1 index, Observed species index, and Simpson index. Beta diversity allows comparison of species diversity among samples, which was analyzed in this study by QIIME (v1.8.0) software based on weighted Unifrac distance (considering sequence abundance) and unweighted Unifrac distance (not considering sequence abundance) with Principal Coordinates Analysis (PCoA). Linear discriminant analysis (LDA) was performed by Linear discriminant analysis Effect Size (LEfSe) software (https://huttenhower.sph.harvard.edu/lefse) to filter for taxonomic units that yielded significant differences between groups at the genus level (*p* < 0.05 with LDA values > 3.0). A permutational multivariate analysis of variance (PERMANOVA) was performed using the *vegan* package in the R 3.4.1 software to decompose the total variance, analyze the degree of explanation of the sample differences by the different grouping factors, and perform significance tests.

Random Forest (RF) classification in R version 3.4.1 predicted RHI MSM or ART MSM using a 70 − 30 split for training and testing, enhancing accuracy via 10-fold cross-validation. The model used crucial variables, and ROC curves were built with AUC calculated using the pROC package.

### Statistical analysis

Statistical analyses used R software (R 3.4.1). Group comparisons: t-tests/ one-way analysis of variance (ANOVA) for normal, Wilcoxon/Kruskal-Wallis for non-normal, and Chi-squared/Fisher’s exact for categorical. Variables’ normality assessed via Shapiro-Wilk test. Spearman’s rank correlation explored relationships, Significance level α = 0.05.

## Results

### General demographic data

In this study a total of 120 participants consisting of 30 uninfected MSM, 30 recent HIV-1 infection MSM, 30 MSM on ART, and 30 non-MSM healthy controls were enrolled. The study group exhibited a median age of 25 years (IQR, 23–28) without notable variations in age or BMI across the groups. The median duration on ART for treated MSM was 18.50 months (IQR, 10.75–29.75) and 96.67% (*n* = 29) of ART-treated MSM had undetectable viral loads (< 50 copies/mL). A majority, 63.33% of MSM on ART, were receiving NNRTI-based regimens, while the remainder were on PI-based and INSTI-based regimens. CD4^+^ T cell counts were evaluated in the three groups, excluding healthy controls. In the RHI MSM group, CD4^+^ T cell counts were markedly lower than uninfected MSM and MSM on ART, with no significant difference between the latter two groups. The median time since HIV-1 diagnosis was 19.50 months (IQR, 14.00–30.50) in the MSM on ART group (Table 1).

**Table 1:** General demographic data

Covariate	Uninfected MSM(*n* = 30)	RHI MSM(*n* = 30)	MSM on ART(*n* = 30)	Non-MSM healthy controls(*n* = 30)	*P*-value
Age (years, median (IQR))	24.50(22.00-25.75)	25.50(23.00-31.00)	25.50(22.25-31.00)	24.00(23.00-26.75)	0.557a
BMI (kg/m^2^, mean (SD))	22.05(1.74)	22.29(2.70)	22.21(2.56)	22.47(2.09)	0.913b
Sexual orientation (%)					< 0.001c
Homosexual	25(83.33%)	23(76.67%)	28(93.33%)	0(0.00%)	
Heterosexual	0(0.00%)	0(0.00%)	0(0.00%)	30(100.00%)	
Bisexual	2(6.67%)	4(13.33%)	2(6.67%)	0(0.00%)	
Not sure	3(10.00%)	3(10.00%)	0(0.00%)	0(0.00%)	
CD4 cell count (cells/µL, mean (SD))	631.47(202.24)	405.35(213.69)	662.90(227.95)	-	< 0.001b
Viral load < 50 copies/mL (%)	-	0(0.00%)	29(96.67%)	-	-
Viral load(copies/mL, M(P25,P75))	-	19013.00(9394.25,55925.00)	0.00(0.00,0.00)	-	< 0.001d
Time since HIV-1-diagnosis (months, median (IQR))	-	-	19.50(14.00-30.50)	-	-
ART duration (months, median (IQR))	-	-	18.50(10.75-29.75)	-	-
ART regimen (%)					-
NNRTI-based	-	-	19(63.33%)	-	
PI-based	-	-	6(20.00%)	-	
INSTI-based	-	-	5(16.67%)	-	

Abbreviations: ART, antiretroviral therapy; PI, protease inhibitors; HIV, human immunodeficiency virus; RHI, Recent HIV-1 infection; NNRTI, nonnucleoside reverse transcription inhibitors; IQR, interquartile range; INSTI, integrase strand transfer inhibitors

a: Kruskal-Wallis test

b: analysis of variance (ANOVA)

c: Fisher’s Exact test

d: Mann-Whitney U test

### Fecal microbiota profiling

The study examined the intestinal microbiota profiles of all participants by 16S rDNA gene sequencing in 120 individual stool samples, one per patient. Post-merging and filtering, 6,885,776 high-quality sequence reads remained, averaging 57,381 sequences per sample for analysis. Using a 97% similarity clustering approach on 1,442,505 tags, we identified 765 operational taxonomic units (OTUs). Uninfected MSM: 626 OTUs, RHI MSM: 681, MSM on ART: 562, General population: 650 OTUs. 482 OTUs (63%) were shared across these groups (Supplementary Fig. 1).

**Fig. 1:**
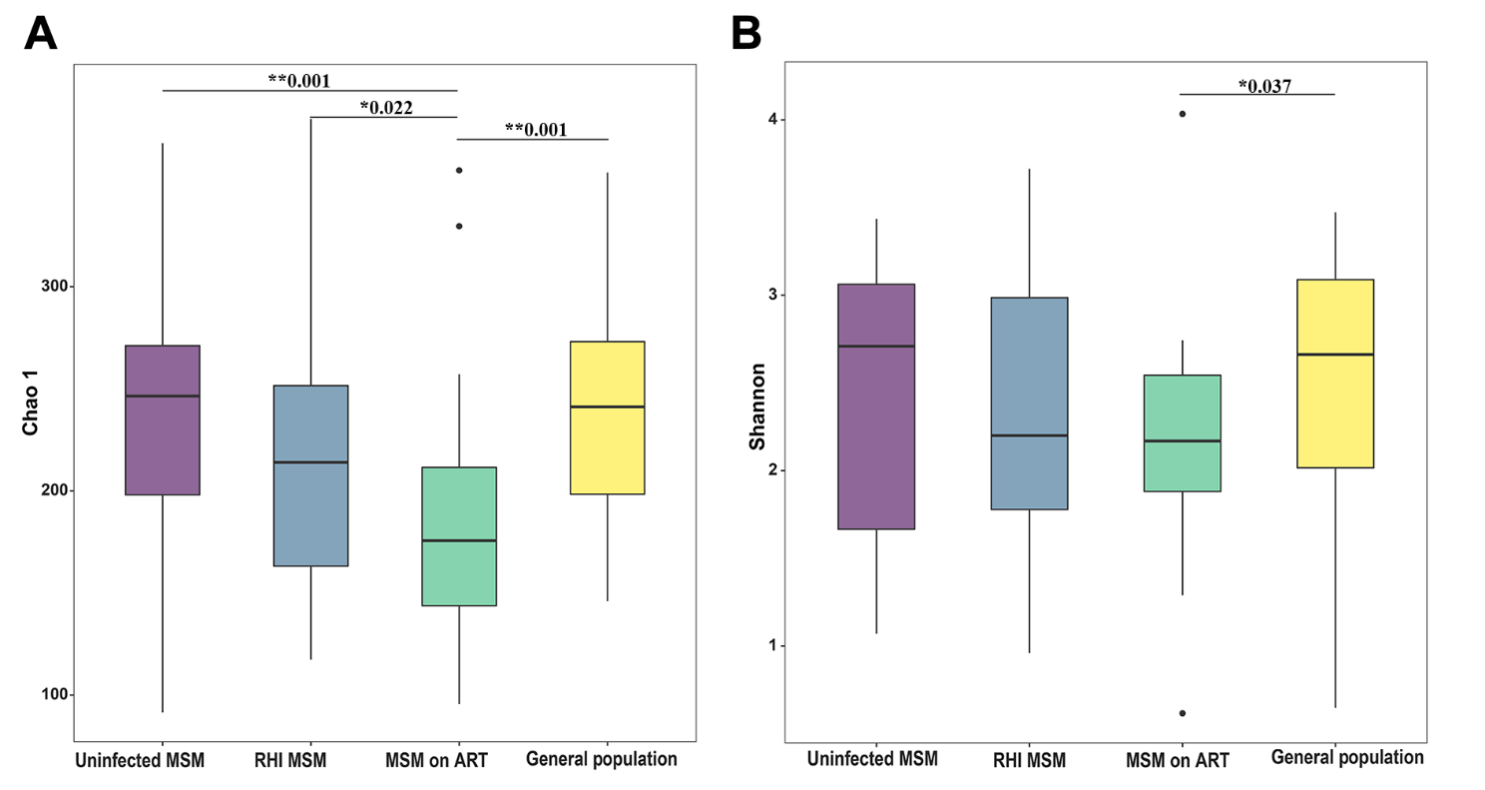
α diversity of bacterial species among four groups: uninfected MSM (*n* = 30), RHI MSM (*n* = 30), MSM on ART (*n* = 30), and General population (*n* = 30). (**A**) α diversity assessed by Chao 1 diversity. (**B**) α diversity assessed by Shannon diversity. *p < 0.05, **p < 0.001

Alpha diversity metrics (Chao 1 and Shannon index) showed that MSM on ART exhibited the lowest biodiversity within the four groups. Alpha diversity was comparable among uninfected MSM, RHI MSM, and the general population, indicating no notable variations (*P* > 0.05, Fig. 1).

**Fig. 2:**
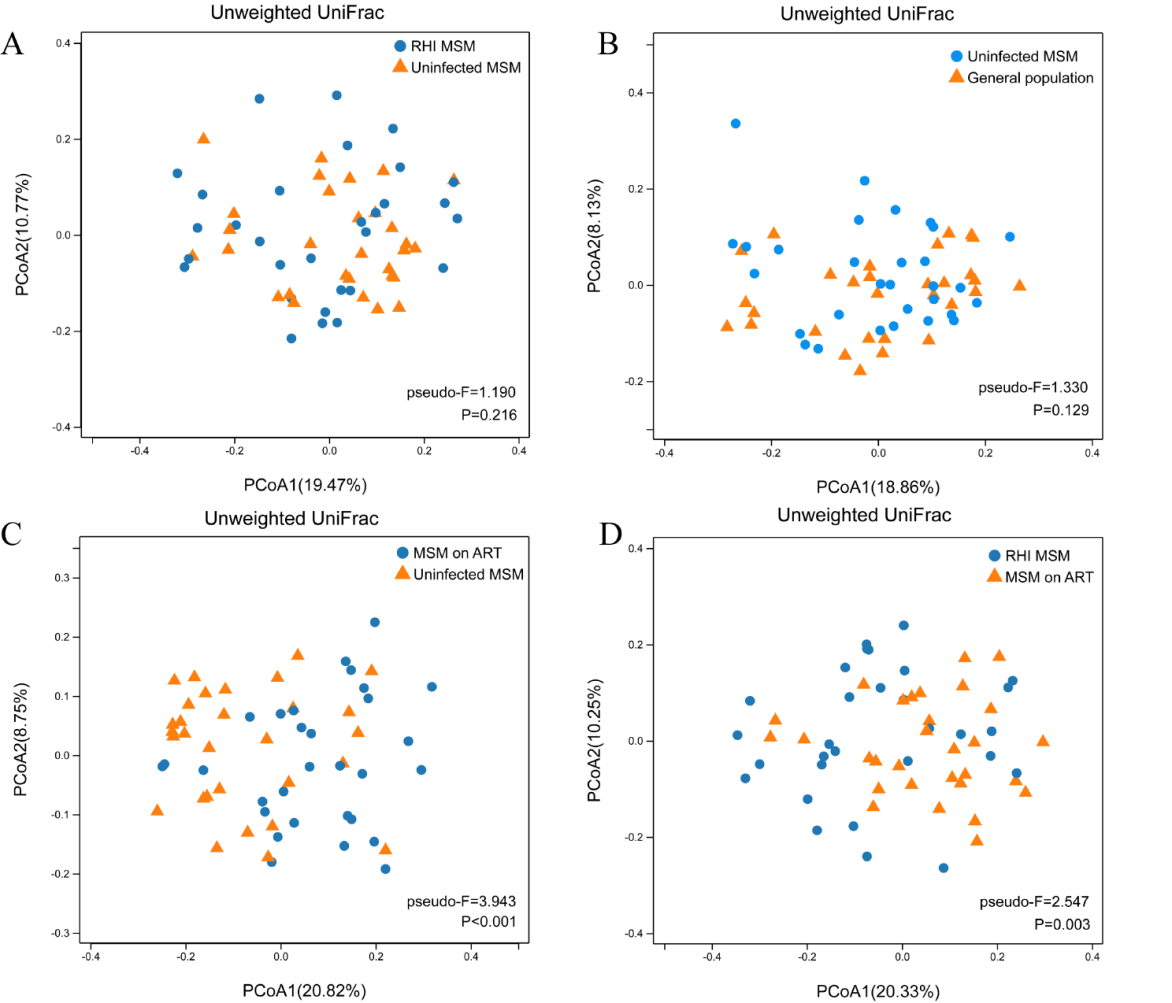
β diversity of bacterial species among four groups: uninfected MSM (*n* = 30), RHI MSM (*n* = 30) ,MSM on ART (*n* = 30)and general population (*n* = 30). (**A**) Comparing β diversity between RHI MSM and uninfected MSM. (**B**) Comparing β diversity between uninfected MSM and general population. (**C**) Comparing β diversity between MSM on ART and uninfected MSM. (**D**) Comparing β diversity between RHI MSM and MSM on ART

Regarding the intestinal microbiota β-diversity, unweighted UniFrac PCoA demonstrated that MSM on ART had a different pattern of clustering when compared with uninfected MSM and RHI MSM (*pseudo-F*: 3.943, *P* < 0.001 and *pseudo-F*: 2.547, *P* = 0.003, respectively) (Fig. 2C and D), suggesting a significant effect of ART on microbiota structure. However, no separation in community structure was observed between RHI MSM and uninfected MSM (*pseudo-F*: 1.190, *P* = 0.216), general population, and uninfected MSM (*pseudo-F*: 1.330, *P* = 0.129) (Fig. 2A and B). Furthermore, similar findings were obtained based on weighted UniFrac distance. (Supplementary Fig. S2).

**Fig. 3:**
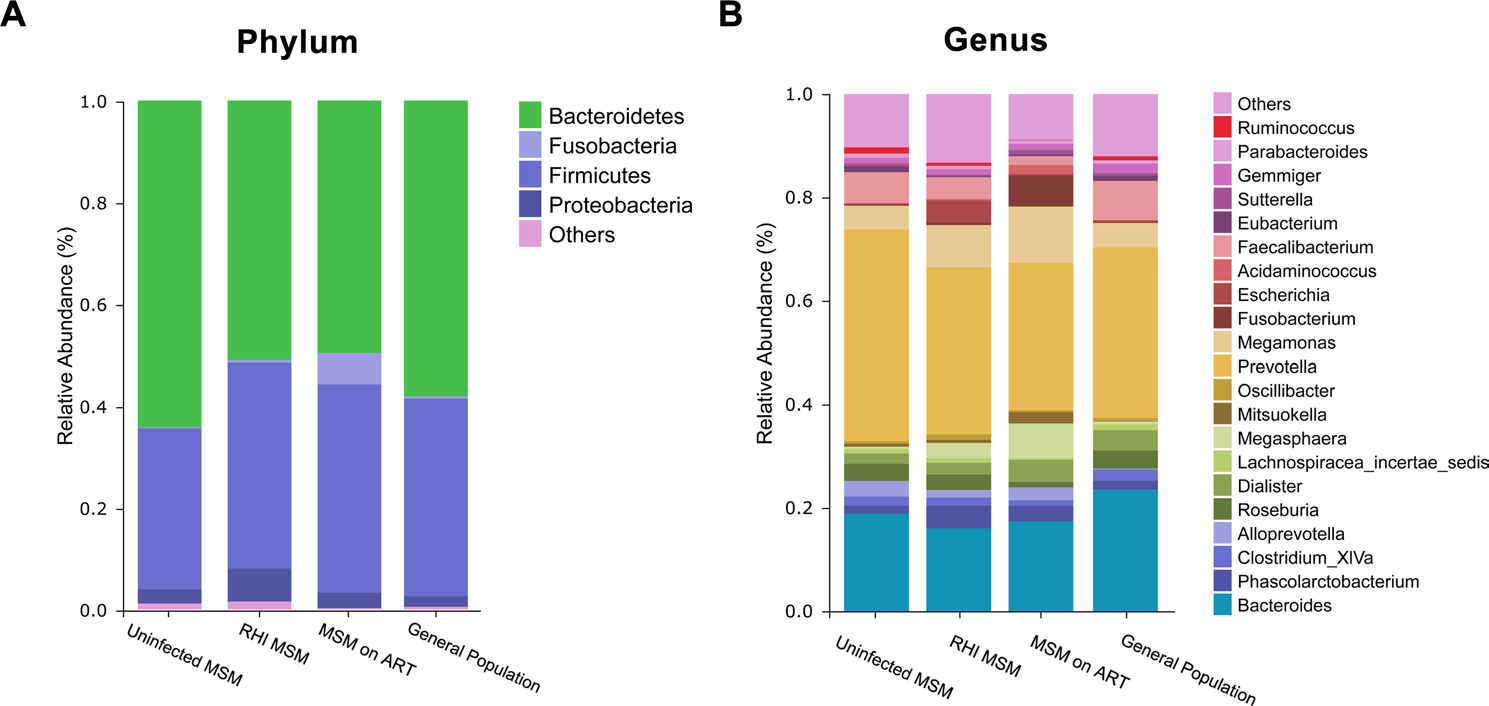
Community structure. (**A**) Barplots illustrating key bacterial Phyla across the four groups. (**B**) Barplots illustrating key bacterial Genus across the four groups

Figure 3A and 3B show average bacterial phyla and genera abundances in their respective groups. Proteobacteria, Bacteroidetes, Fusobacteria, and Firmicutes collectively constituted 98% of fecal samples in each group, establishing them as the predominant phyla. At the taxonomic level of genus, *Prevotella* and *Bacteroides* were the most dominant genus in all four groups.

**Fig. 4:**
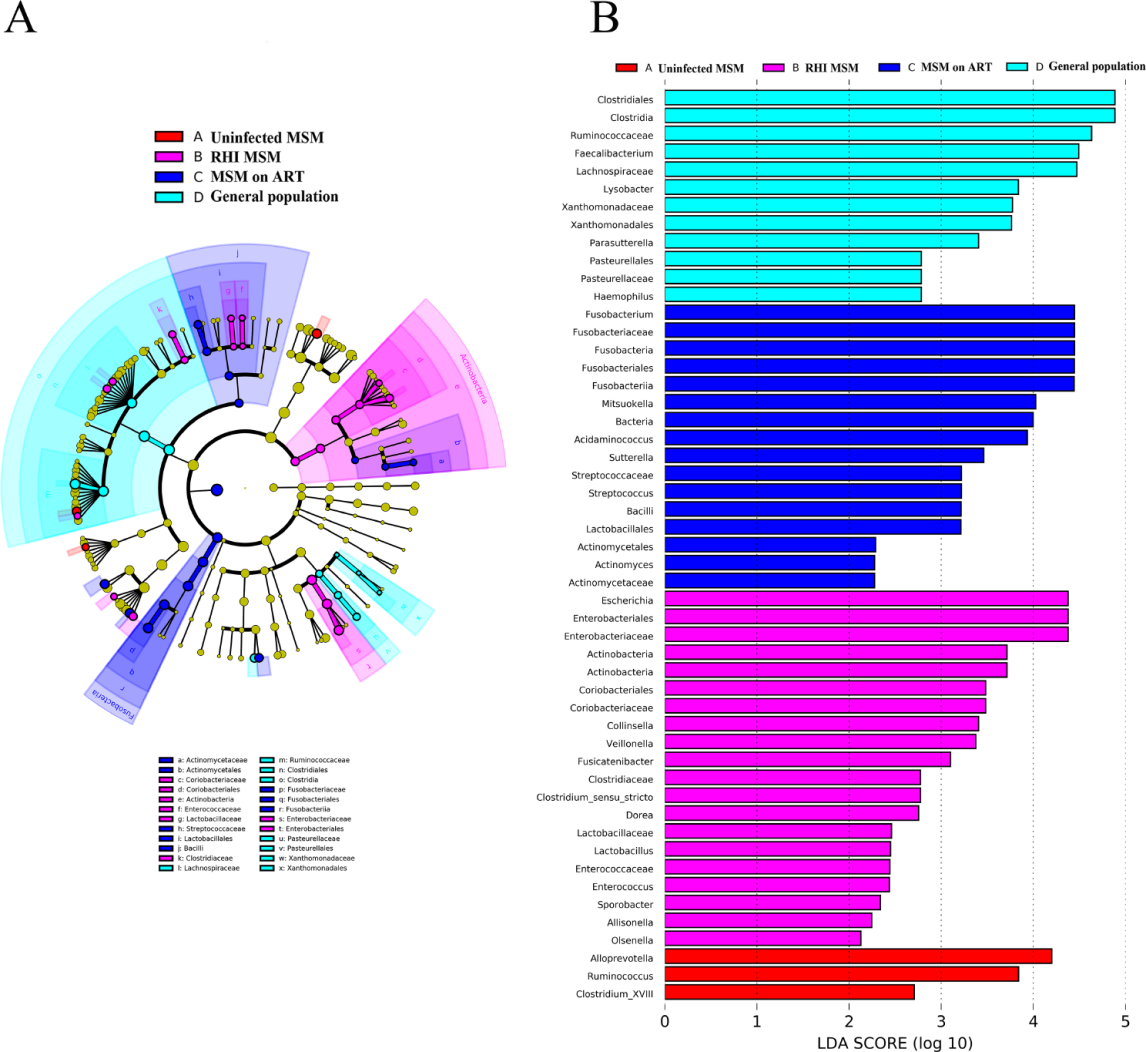
(**A**) The cladogram displays variances in stool microorganism categories, denoted by letters corresponding to differentially abundant taxa. (**B**) LEfSe analysis conducted on the four groups’ intestinal microbiota. Histogram displays significant bacteria (LDA > 2) differences between groups. Different colors indicate different groups in (**A**); taxa with LDA > 2 are highlighted in the histogram in (**B**)

To detect variations in abundant taxa, we conducted linear discriminant analysis (LDA) effect size (LEfSe) analysis on the composition of fecal microbiota across the four groups. LEfSe analysis identified 51 discriminatory features (LDA > 2, *P* < 0.05, Fig. 4A). At genus level, 24 bacterial taxa display different relative abundances among the four groups. MSM on ART showed higher prevalence rates of *Fusobacterium*, *Mitsuokella*, *Acidaminococcus*, *Sutterella*, *Streptococcus*, and *Actinomyces* than the other groups. In RHI MSM, dominant genera include *Escherichia*, *Collinsella*, *Veillonella*, *F usicatenibacter*, *Clostridium_sensu_stricto*, *Dorea*, *Lactobacillus*, *Enterococcus*, *Sporobacter*, *Allisonella*, and *Olsenella*, Uninfected MSM have higher levels of *Alloprevotella*, *Ruminococcus*, and *Clostridium_XVIII.* General population’s fecal microbiome had higher *Faecalibacterium*, *Lysobacter*, *Parasutterella*, and *Haemophilus* levels than other groups. Intestinal microbiota composition and structure differed significantly between MSM on ART and RHI MSM (Fig. 4B).

### Effects of diverse ART regimens on fecal microbiota (in patients with HIV)

**Fig. 5:**
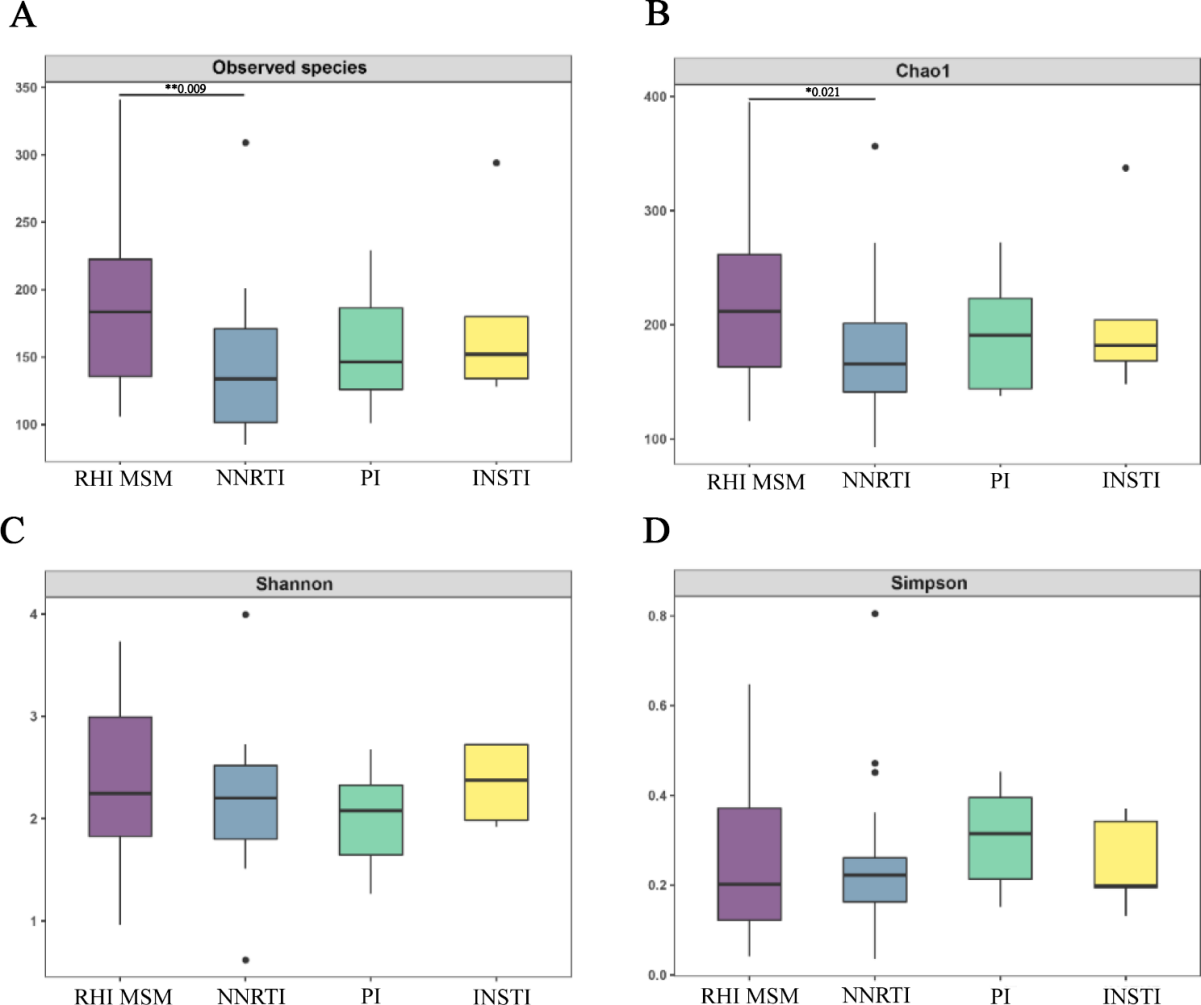
Comparison of intestinal bacterial α diversity between different ART regimens group and untreated group. (**A**) Observed species, (**B**) Chao 1 indices, (**C**) Shannon indices, (**D**) Simpson indices. *p < 0.05, **p < 0.001

**Fig. 6:**
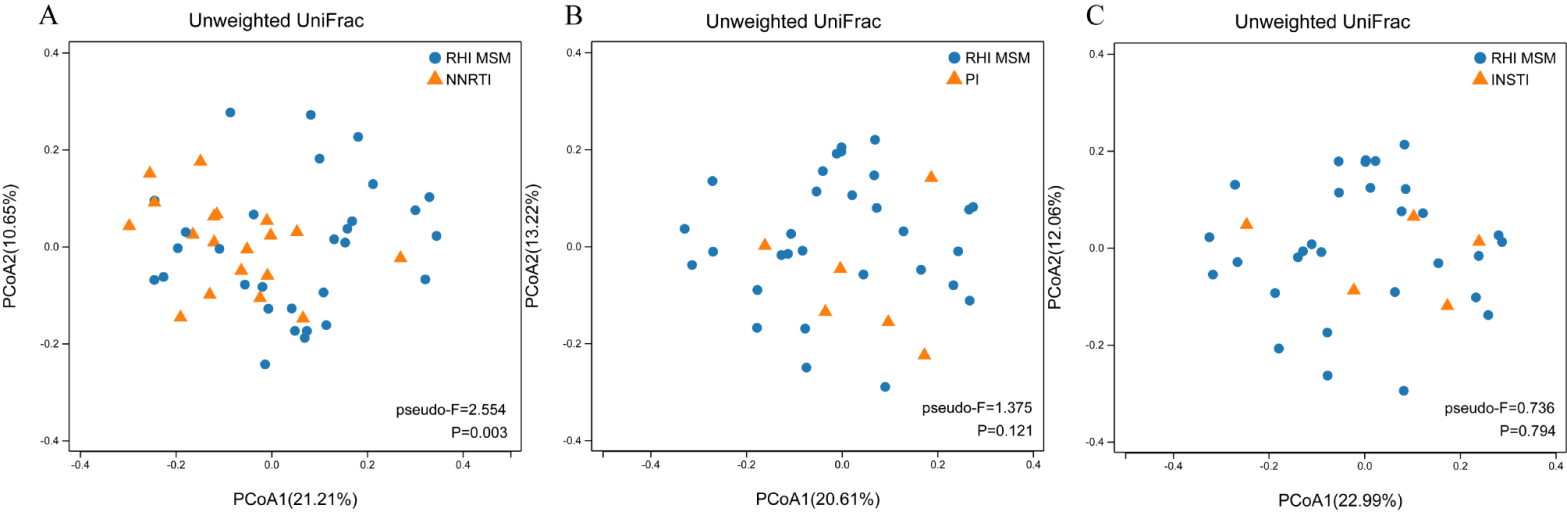
PCoA diagram represents intestinal bacterial beta diversity using unweighted UniFrac distance. (**A**) Evaluating β diversity differences between RHI MSM and NNRTI groups. (**B**) Evaluating β diversity between RHI MSM and PI. (**C**) Evaluating β diversity between RHI MSM and INSTI.

**Fig. 7:**
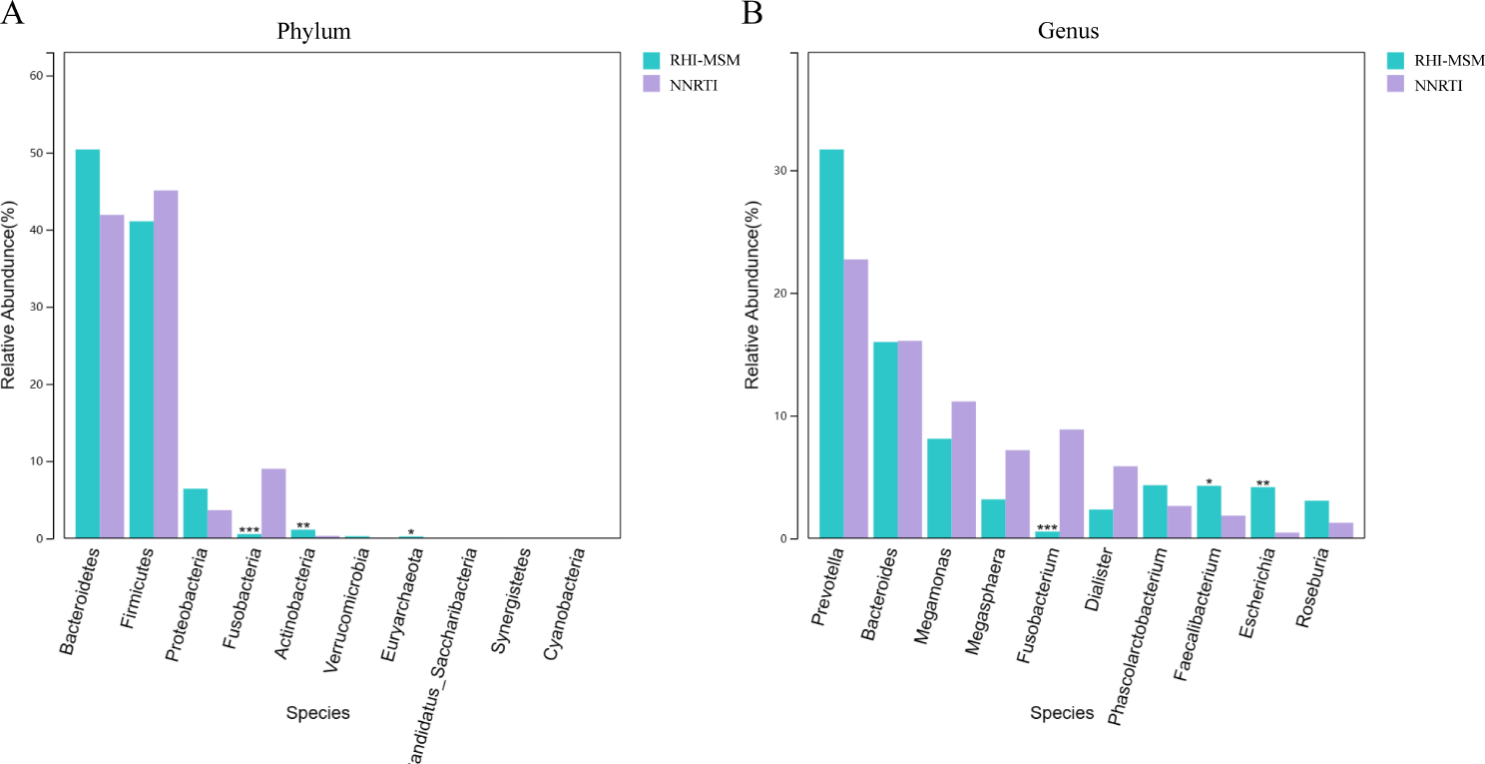
Analyzing intestinal microbiota abundance in varied medication regimens at Phylum (**A**) and Genus (**B**) levels. *p < 0.05, **p < 0.001, ***p < 0.0001

We categorized our ART cohort by the third drug used: NNRTI, PI, or INSTI, enabling a focused analysis of their respective roles. Surprisingly, upon stratification by ART regimens, only the NNRTI-based group showed a significant reduction in richness (Chao1: *P* = 0.006; Fig. 5), but not evenness indexes (Shannon Fig. 5), compared with RHI MSM. Furthermore, similar findings were observed at β diversity. Unweighted UniFrac PCoA showed that the microbiota structure of the NNRTI-based group differed from that of RHI MSM (Fig. 6). No separation in community structure was seen between the PI-based group, the INSTI-based group, and the RHI MSM (Fig. 7).

Analyzing top 10 species, we compared the differences in the relative abundance of key species in four groups. At phylum level, NNRTI-based regimens were linked to a notably higher presence of Fusobacteria (0.53% vs. 9.00% *P*<0.001), and a reduced presence of Actinobacteria (1.09% vs. 0.30% *P* = 0.002) and Euryarchaeota (0.22% vs. 0.00% *P* = 0.026) compared with RHI MSM. At the genus level, NNRTI-based regimens significantly increased the genus of *Fusobacterium* (0.53% vs. 8.86% *P*<0.001) and reduced the *Faecalibacterium* (4.27% vs. 1.84% *P* = 0.041) and *Escherichia* (4.16% vs. 0.45% *P* = 0.009) genera (Fig. 7).

## Examining intestinal microbiota correlations with clinical factors

### Microbial translocation and inflammation markers in plasma

**Fig. 8:**
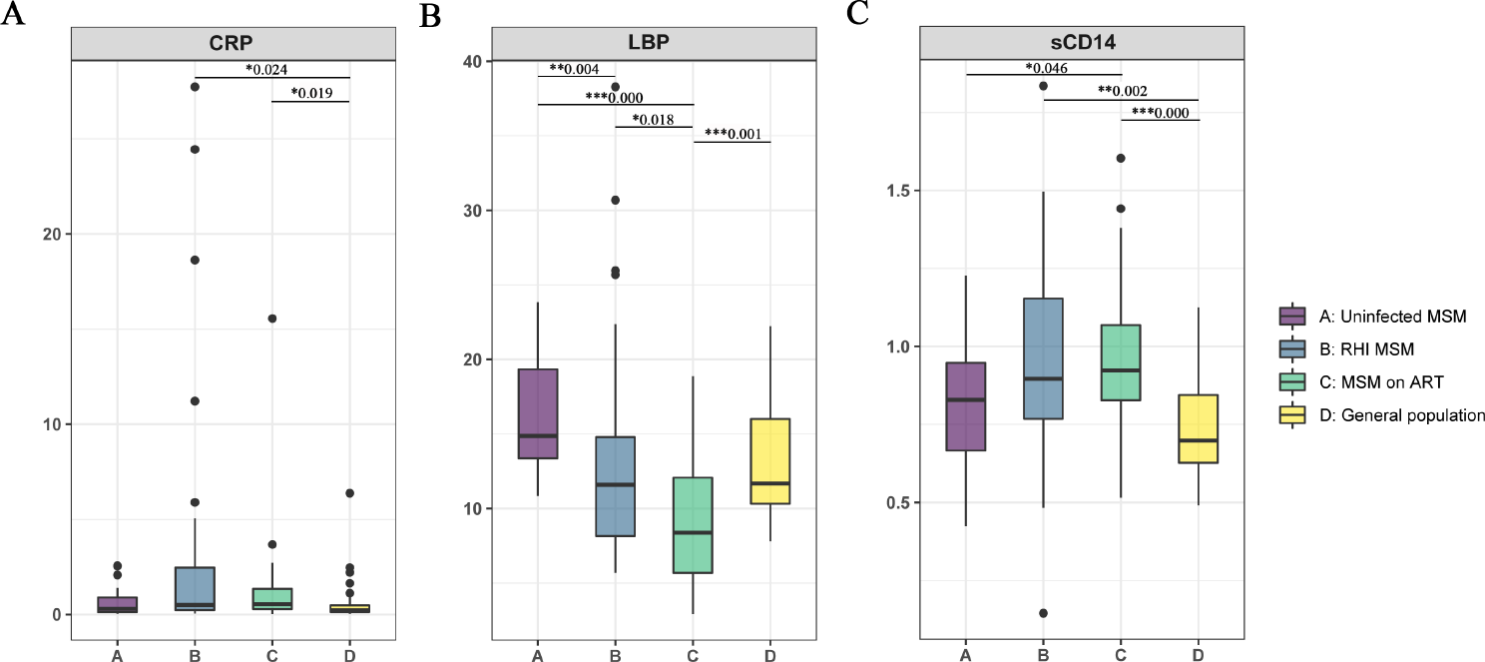
Plasma markers for microbial translocation and inflammatory factors were assessed across the four groups. (**A**) C-reactive protein, CRP, (**B**) lipopolysaccharide binding protein, LBP, (**C**) soluble CD14, sCD14.*p < 0.05, **p < 0.01, ***p < 0.0001

**Fig. 9:**
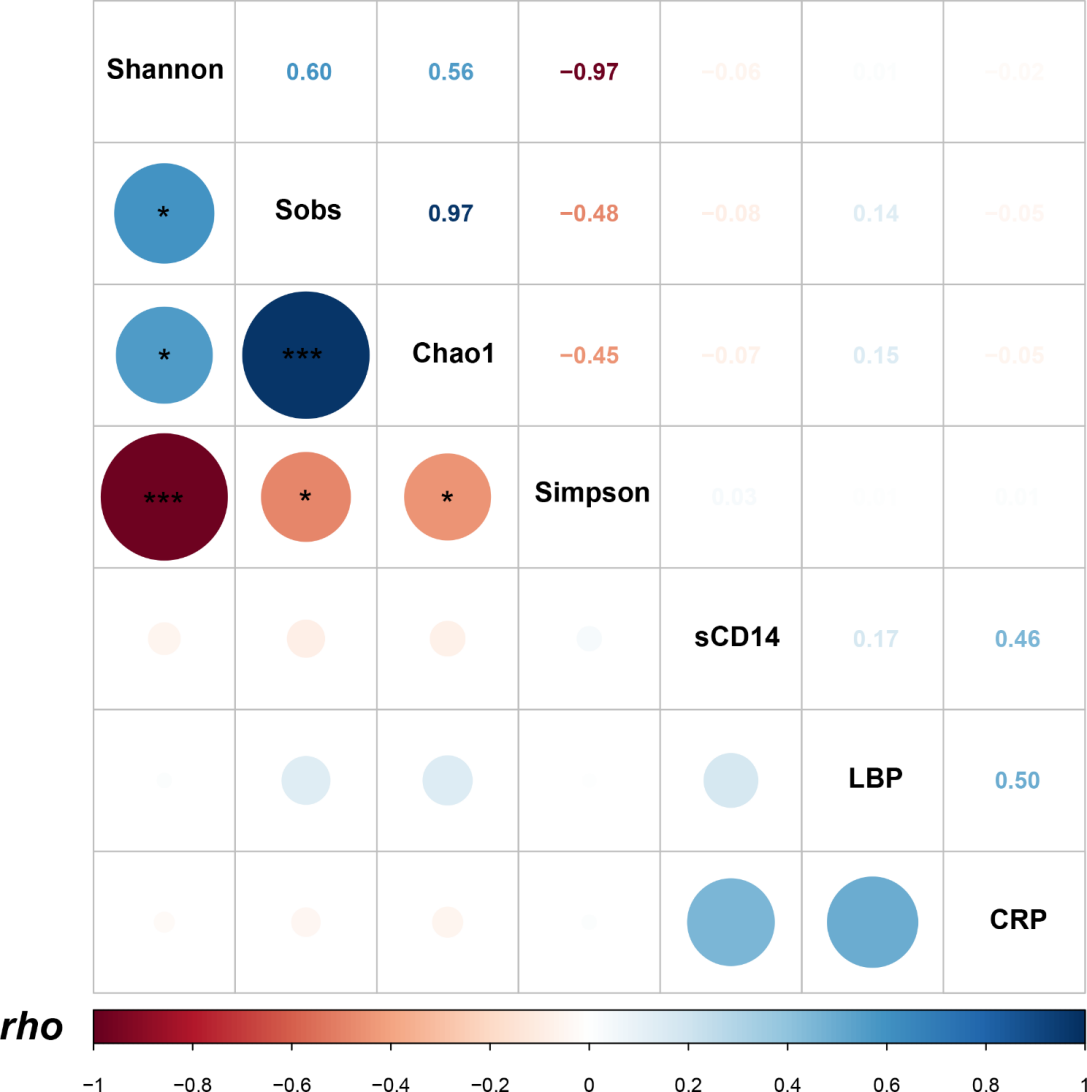
Matrix diagram of correlation coefficient between α diversity and microbial translocation markers and inflammatory factors. * *p* < 0.05, ** *p* < 0.001, *** *p* < 0.0001

As an inflammation biomarker, plasma CRP levels were examined, and it was found that both RHI MSM and MSM on ART showed higher levels when compared to the general population (ALL *P* < 0.05, Fig. 8A). No significant differences were observed between RHI MSM and MSM on ART (*P* > 0.05, Fig. 8A). Additionally, plasma concentrations of sCD14 and LBP, markers indicating microbial translocation, were evaluated (Fig. 8B and C). Elevated sCD14 levels were evident in MSM on ART, distinguishing them from uninfected MSM and the general population (*P* < 0.05, *P* < 0.0001, Fig. 8C), whereas no significant distinction was observed between uninfected MSM and the general population (*P* = 0.117, Fig. 8C). Additionally, MSM on ART exhibited substantially lower LBP levels in comparison to uninfected MSM (*P* < 0.001, Fig. 8B) and RHI MSM (*P* < 0.05, Fig. 8B). Significant differences in plasma LBP levels were found between uninfected MSM and RHI MSM (*P* < 0.001, Fig. 8B). Interestingly, no associations were found between the plasma CRP, sCD14, and LBP levels (*P* > 0.05, Fig. 9). Moreover, no significant statistical correlation was identified between these microbial translocation and inflammation markers and alpha diversity (ALL *P* > 0.05, Fig. 9).

### Intestinal Microbiota and clinical indicators

**Fig. 10:**
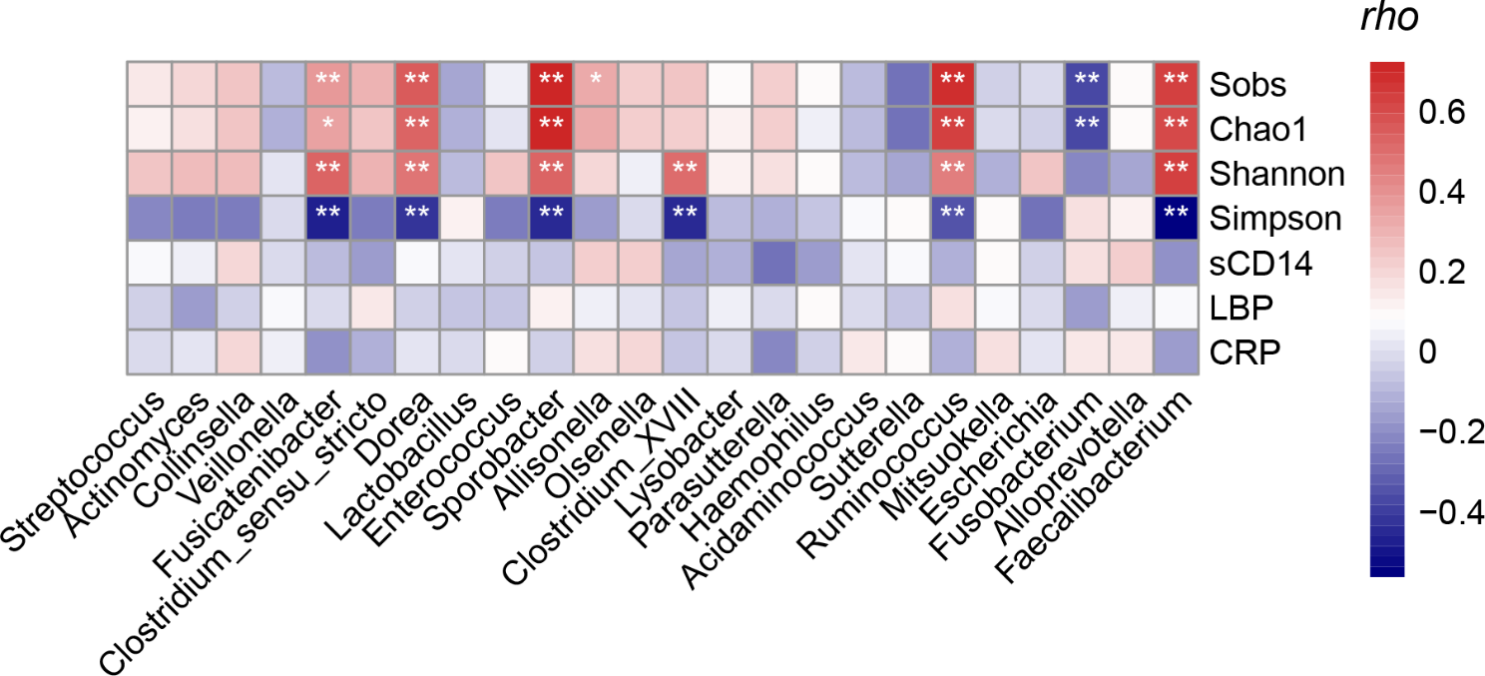
Heatmap of the relationship between differential bacteria and microbial translocation markers and inflammatory factors. sCD14, soluble CD14; LBP, lipopolysaccharide binding protein; CRP, C-reactive protein. * *p* < 0.05, ** *p* < 0.001

The study analyzed gut microbiota’s links to translocation markers, inflammation, and alpha diversity. Certain genera displayed negative correlations with translocation and inflammation markers, yet lacked statistical significance. However, the relative abundance of *Faecalibacterium*, *Ruminococcus*, *Clostridium_XVII*I, *Sporobacter*, *Dorea*, and *Fusicatenibacter* was positively correlated with fecal microbiome alpha diversity. Conversely, we found a significant inverse relationship between *Fusobacterium*’s relative abundance and α diversity (Fig. 10).

### Intestinal microbiome: non-invasive HIV diagnosis for MSM

**Fig. 11:**
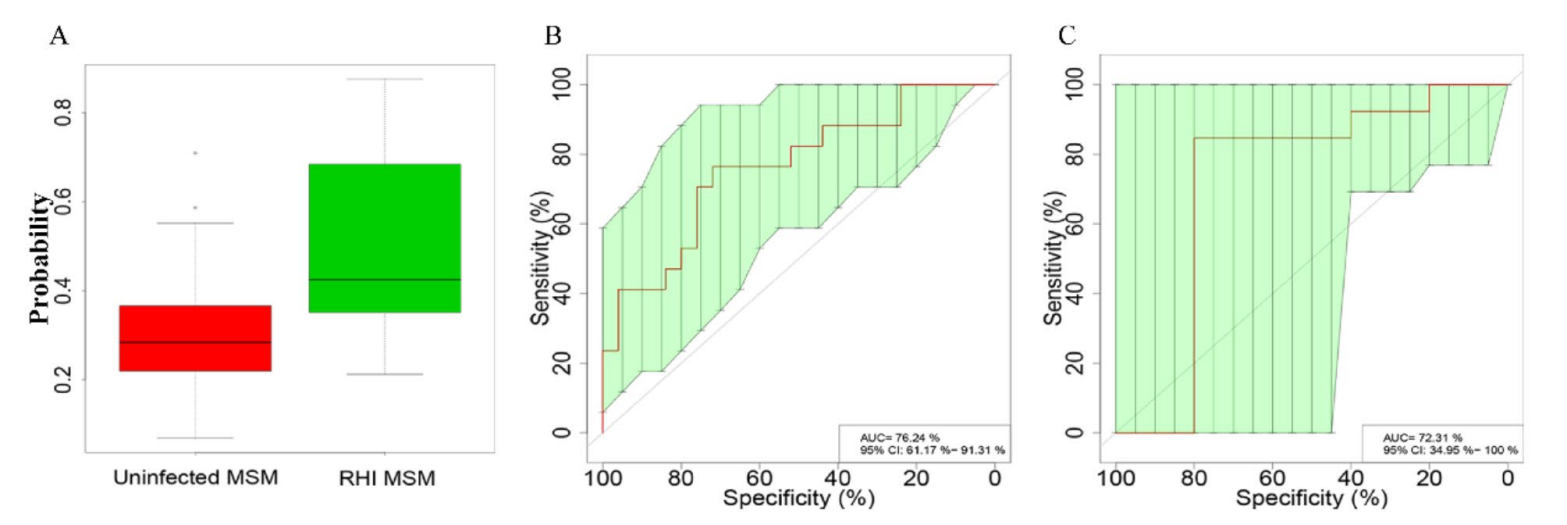
Non-Invasive HIV Infection Diagnosis in MSM Through Intestinal Microbiome Model. (**A**) The POD value exhibited a significant rise in RHI MSM in contrast to HIV-uninfected MSM counterparts. (**B**) Cross-validated ROC analysis used 5 OTUs in training with random forest models. The AUC was 76.24% (95% CI: 61.17 − 91.31%. (**C**) ROC analysis on the test set showed an AUC of 72.31% (95% *CI*: 34.95 − 100%)

We built a random forest classifier model with 30 HIV-uninfected MSM and 30 RHI MSM samples to assess intestinal microbial markers’ diagnostic potential in HIV infection among MSM. Ten-fold cross-validation and the ROC curve for distinguishing RHI MSM from HIV-uninfected MSM were developed. Finally, five OTUs (*Escherichia, Roseburia, Megasphaera, Ruminococcus*, and *Oscillibacter*) were selected as the optimal markers to identify differences between the two groups, and the AUC was 76.24% (95% *CI*: 61.17 − 91.31%) (Fig. 11B). Subsequently, we computed the Probability of Disease (POD) index for the training set utilizing the five selected OTUs. The POD index demonstrated a notable elevation in RHI MSM in comparison to HIV-uninfected MSM (Fig. 11A).

The test set was employed to validate the diagnostic effectiveness of microbial biomarkers for HIV infection. The AUC was 72.31% (95% *CI*: 34.95 − 100.00%) (Fig. 11C), which highlighting intestinal microbiota’s potential in identifying HIV infection. In summary, the results emphasize the possibility of utilizing intestinal microbiota biomarkers for non-invasive detection of HIV infection in MSM.

## Discussion

The HIV prevention and control situation among MSM, a key demographic for HIV infection, is currently a cause for concern. Research underscores the vital role of gut microbiota in HIV immunopathogenesis, intricately connected to the emergence and advancement of HIV-related comorbidities. This study analyzed and compared intestinal microbiota composition and diversity in uninfected MSM, RHI MSM, MSM on ART, and non-MSM healthy individuals using 16S rDNA amplicon sequencing technology. The findings showed comparable intestinal microbiota diversity in MSM and non-MSM populations, with distinct alterations in the specific microbial composition within these groups. Following HIV infection, a relatively rapid onset of mild intestinal microbiota dysbiosis is observed in MSM (Fig. 1). Notably, the effective administration of ART fails to alleviate this dysbiosis among HIV-positive MSM, and in some cases, appears to exacerbate it, suggesting a possible direct impact of ART medications on specific intestinal microbial communities (Fig. 5). These results deepen our insight into the intestinal microbiota features of HIV-infected MSM individuals.

A recent study indicated that intestinal microbiota might impact HIV infection risk and disease progression in MSM [29]. In this research, the intestinal microbiota diversity and community structure were alike in both the uninfected MSM group and the healthy non-MSM group. However, concerning specific species composition, it seems that the microbiota in MSM may be shifting towards a pattern that is potentially more pathogenic. In the uninfected MSM group, the phyla Bacteroidetes and Proteobacteria, and genus *Prevotella* showed increased abundance. Conversely, the phylum Firmicutes and the genus *Bacteroides* decreased during the observation. These findings are in line with previous research results [12, 30, 31].

An investigation involving naive macaques demonstrated an intestinal microbiota composition with a lower ratio of *Prevotella* to *Bacteroides* and reduced abundance of the phylum Firmicutes with mucosal transmission of simian immunodeficiency viruses (SIVs) [32]. The genus *Prevotella*, as a member of the intestinal microbiota, has been established to exhibit pro-inflammatory effects, whereas the genus *Bacteroides* has been demonstrated to possess anti-inflammatory properties by enhancing the reactivity of T regulatory cells [22]. During anal intercourse, the receptive partner (the individual being penetrated) faces higher HIV infection risk than the insertive partner (the individual doing the penetration). Additionally, various characteristics of different same-sex sexual behaviors may lead to varying degrees of alterations in the intestinal microbiota and inflammatory states. Receptive anal intercourse poses a higher HIV risk than insertive, as per research [33]. Hence, conducting thorough research is crucial to understanding how alterations in intestinal microbiota among MSM may heighten HIV susceptibility. To analyze acute HIV’s impact on intestinal microbiota and ART, four participant groups were recruited. Matching criteria, including age and BMI, were applied to ensure comparability between the four groups. Findings show decreased α-diversity in intestinal microbiota of RHI MSM compared to uninfected MSM. Furthermore, a shift was noticed with a decrease in beneficial bacteria and a rise in potentially harmful bacteria in their intestinal microbiota, indicating a mild disruption in intestinal microbiota composition following HIV infection. Consistent with prior research [34–37], our findings support the observation of elevated levels of *Proteobacteria*, especially within the *Enterobacteriaceae* family, in treated and untreated chronic HIV-infected individuals compared to HIV-uninfected controls. However, it’s important to highlight that within the HIV on ART group, a notable reduction in the levels of the *Enterobacteriaceae* family was detected, suggesting that ART may partially reverse the changes in microbiota composition.

To investigate the influence of ART on the gut microbiota of HIV-infected MSM, we divided ART-treated individuals into three groups: NNRTI, PI, and INSTI, for further analysis. In the NNRTI group, intestinal microbiota diversity decreased significantly, differing from RHI MSM; in contrast, the PI and the INSTI groups did not exhibit similar findings. This indicates that NNRTI-class medications may directly influence specific intestinal microbiota, thereby affecting the microbial ecological balance, aligning with previous research [38]. It is worth noting that in this study, the individuals in the NNRTI group were all taking EFV, a non-nucleoside antiviral medication. This indicates that the observed microbial dysbiosis is likely a result of EFV’s antimicrobial effects. Previous study [39] have indicated that individuals undergoing treatment with INSTI-based regimens exhibit intestinal microbiota diversity and inflammation levels comparable to those observed in uninfected individuals. INSTI-based therapies hold potential in addressing gut microbiota imbalances in HIV patients, offering a promising intervention avenue. Our study also yielded similar findings, and the underlying mechanism may be associated with the ability of INSTI-class antiviral drugs to induce a significant reduction in pre-viral DNA, thereby promoting rapid immune reconstitution [40]. Hence, in future treatment regimen adjustments, there could be a greater consideration for the inclusion of INSTI-class medications.

In this study, the RHI MSM group exhibited elevated levels of sCD14, a marker indicating microbial translocation, in comparison to the uninfected MSM group (Fig. 8C). This suggests that HIV infection may lead to intestinal epithelial barrier disruption and alterations in intestinal permeability, potentially allowing for the translocation of microbiota or their metabolic products into the bloodstream. CRP levels within the three MSM groups exhibited a trend similar to that of sCD14 (Fig. 8A and B); this provides additional evidence for the persistence of chronic gastrointestinal inflammation following HIV infection, despite the use of ART. The correlation analysis revealed a positive association between sCD14 and CRP, indicating a significant positive correlation between these two biomarkers. Our study found a strong negative correlation between *Fusobacterium* genus presence and intestinal microbiota diversity. This implies that changes in the *Fusobacterium* genus could be a crucial factor contributing to the significant decrease in intestinal microbiota diversity observed in MSM individuals. Therefore, targeting the *Fusobacterium* genus could be a potential focus for future therapeutic and intervention strategies.

Based on intestinal microbiota sequencing data, this study employed a random forest model with 10-fold cross-validation at the OTU level, identifying 5 OTUs as optimal biomarkers for HIV infection with an AUC of 76.24% in ROC analysis. This highlights the importance of selecting high-accuracy HIV infection diagnostic biomarkers, which could play a crucial role in early detection and treatment.

This study has the following limitations: (1) this study, being cross-sectional in design, cannot establish definitive causal relationships; (2) this study utilized 16S sequencing, allowing taxonomic classification only at the genus level. To achieve greater taxonomic resolution, future investigations may consider employing a shotgun metagenomics study; (3) Due to stigma and concerns for loss of privacy and participant safety, we did not collect information on sexual preferences. Therefore, we are unable to assess potential confounding resulting from associations between sexual practices and microbiome differences; (4) Because of the limitations of our sample size and the low number of subjects with the other ART, our results regarding the differences between ART regimens still need to be extrapolated by increasing the sample size.

## Conclusion

In summary, MSM individuals may exhibit mild intestinal microbiota dysbiosis shortly after HIV infection, and ≥ 6 months of ART does not ameliorate this dysbiosis; instead, it exacerbates the disruption. When comparing ART regimens, NNRTI-based treatment significantly altered intestinal microbiota composition, indicating a direct impact of NNRTI-class drugs. The exploration of diagnostic biomarkers for HIV infection using the random forest model has indicated that the gut microbiota holds potential for non-invasive diagnosis of recent HIV infections. It may be considered a supplementary diagnostic method in the future.

### Electronic supplementary material

Below is the link to the electronic supplementary material.

Supplementary Material 1

## Data Availability

Sequence data that support the findings of this study have been deposited in the Sequence Read Archive (SRA) with the primary accession code PRJNA1028262. (https://www.ncbi.nlm.nih.gov/search/all/?term=PRJNA1028262)
