# Supplementary material for: Characterization of the intestinal microbiota in MSM with HIV infection
Source: BMC Microbiol. 2024 Jun 3;24:192. doi: 10.1186/s12866-024-03351-z (PMC11145808; doi:10.1186/s12866-024-03351-z)
Supplement: Supplementary file 1 — Supplementary Material 1 [file 12866_2024_3351_MOESM1_ESM.docx]

Supplementary Material

## Supplementary Figures


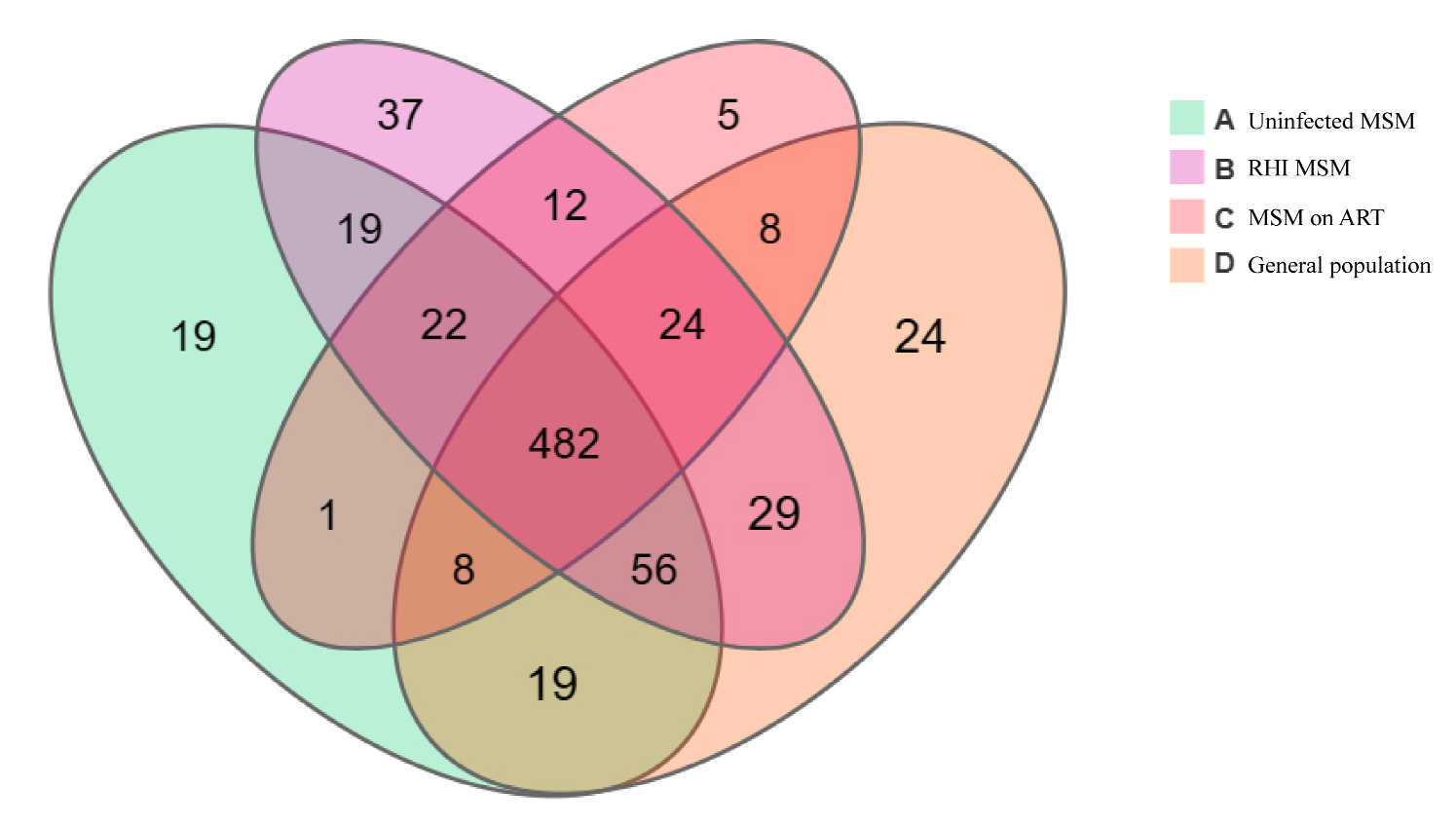


**Supplementary Figure 1.** OTU Venn diagram

**
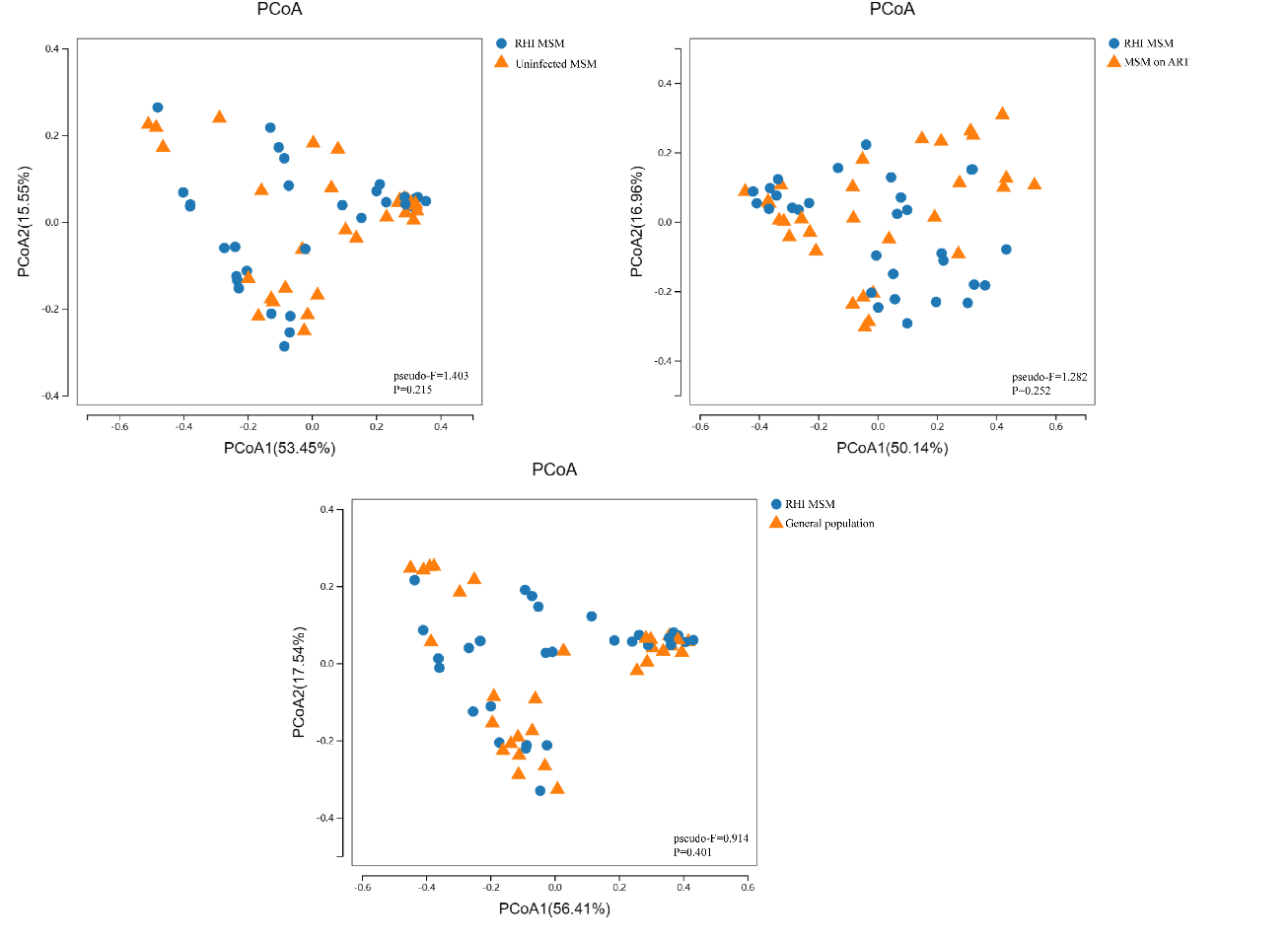
**

**Supplementary Figure 2.** β diversity of bacterial species among four groups: uninfected MSM (n = 30) 、RHI MSM (n= 30) 、MSM on ART (n = 30), and general population (n=30).
